# Three-Dimensional Imaging Sensor-Based Non-Contact Measurement of Neonatal Head Circumference in Incubators

**DOI:** 10.3390/s25061869

**Published:** 2025-03-18

**Authors:** Khin Dagon Win, Kikuhito Kawasue, Masatoki Kaneko

**Affiliations:** 1 Faculty of Engineering, University of Miyazaki, Miyazaki 889-2192, Japan; dagon@cc.miyazaki-u.ac.jp; 2 Faculty of Medicine, University of Miyazaki, Miyazaki 889-1692, Japan; mkaneko@med.miyazaki-u.ac.jp

**Keywords:** newborns, head circumference, mat, 3D imaging sensor, incubator

## Abstract

**Highlights:**

**What are the main findings?**

- A non-contact method for measuring neonatal head circumference using a 3D imaging sensor was developed.

**What is the implication of the main finding?**

- A top-view 3D imaging system can be used to estimate the head circumferences of neonates without direct contact, ensuring safety for their delicate skin. Because some portions of the head may be missing in the top-view images, these missing areas are estimated using mat information. The extracted features and head surface values contribute to the head circumference measurement. This approach enhances the feasibility of non-contact head circumference measurement, which could improve neonatal monitoring in clinical settings.

**Abstract:**

In Japan, birth rates are declining, but there are a rising number of underweight newborns who require specialized care in neonatal intensive care units (NICUs). Head circumference is an important indicator of brain development for low-birth-weight infants. However, measuring head circumference requires extreme care because low-birth-weight infants have fragile skin. Therefore, a non-contact measurement system using a 3D imaging sensor was developed. Using this system, three-dimensional data for a newborn’s head can be obtained from outside the incubator. Briefly, the images are taken from above the incubator, so there is an area behind the head that cannot be captured by the camera, but the head circumference estimation takes into account the fact that the head is in contact with the mat. The proposed method allows head circumference estimation without touching the newborn. This approach minimizes stress for both the neonate and the nurse and improves efficiency and safety in the NICU.

## 1. Introduction

In Japan, the birth rate is gradually decreasing, but the number of preterm infants is steadily increasing [1,2,3,4]. Premature infants experience frequent aversive procedures, excess handling, disturbance of rest, noxious oral stimulation, noise, and bright light. These are all sources of stress and physiological instability, which may lead to medical complications such as intraventricular hemorrhage and necrotizing enterocolitis. This raises concerns about the need for specialized training for healthcare providers to support the normal development of premature babies. On the other hand, the sensitivity required in neonatal care leads to emotional stress and high workloads for nurses. Therefore, some sensor-based technical advancements have been introduced in NICUs, such as behavior analysis and monitoring systems [5,6,7].

Measuring head circumference is a crucial aspect of monitoring an infant’s brain development. The regular monitoring of head circumference allows healthcare providers to track growth over time, ensuring that infants’ brains are developing appropriately. Establishing accurate measurements can help to identify any deviations from expected growth patterns, enabling early diagnosis and the management of potential complications such as hydrocephalus [8,9,10]. In traditional methods, a soft tape measure is used to confirm the growth patterns of newborns. However, for newborns, especially premature babies with extremely delicate skin [11,12,13], using a traditional soft tape measure presents several challenges. The manual process is susceptible to human error, as variations in positioning and newborn movement can lead to inconsistent readings. Additionally, direct contact increases the risk of infection and may disrupt the newborn’s growth cycle. Healthcare providers must carefully manage multiple factors that can influence measurement accuracy. Consequently, barriers from traditional measurements in practical applications can be significantly large [14,15]. Therefore, there is a need for effective tools and techniques to measure head circumference in a safe and hands-free manner. Head circumference measurement using smartphone images (2D images) has been introduced [16].

In 2D image processing, the distance between the camera and objects can significantly affect measurement accuracy. When the camera is positioned closer to the object, the object appears larger in the captured image, whereas if the camera is farther away, the object appears smaller. Moreover, the perspective of 2D cameras to the images is also important for obtaining a standard shape of newborns. This perspective distortion can lead to errors in size estimation, leading to imprecise measurements. To solve this issue, depth information from 3D imaging sensors is employed to ensure consistency in size estimation across varying distances. In recent years, with the introduction of 3D imaging sensors such as Microsoft Kinect (Microsoft Corporation, Washington, DC, USA), Intel RealSense (Intel Corporation, California, USA), and ASUS Xtion2 (ASUSTeK Computer Inc., Taiwan), it has become possible to obtain three-dimensional (3D) data in a single image, aiding in motion capture and modeling systems [17,18,19,20]. Some human body measurement systems using 3D imaging sensors have recently been introduced [21,22,23,24,25,26,27].

Therefore, a non-contact head circumference measurement method for newborns using a 3D imaging sensor was introduced. Three-dimensional imaging technology captures top views of newborns’ heads, which are then processed using image-processing techniques to measure their circumferences. However, when the camera captures images from the top, some portions of the head are invisible. To address this issue, this study proposes a new approach that takes into account the fact that the head is in contact with the mat. In this study, we introduced the following developments of our measurement system for practical uses in the NICU.

(1) A measurement system using 3D imaging sensors in the NICU is introduced. The camera placement, additional components, and environmental considerations required for development are described in detail.
(2) To obtain precise measurements, a 3D measurement approach is preferred in this system. Therefore, the 3D data transformation method, which converts depth information into real-world coordinates, is also introduced.
(3) The body and head detection technique for YOLOv5 model development, along with its performance in generalization for practical applications, is discussed. The data collection, dataset preparation, and evaluation methods used to determine the model’s accuracy are also highlighted.
(4) The determination of the mat area and the newborn’s head area in world coordinates is discussed. Their role in estimating missing head locations is explained in detail, including the methods used to estimate occluded regions by using a 3D image-processing technique.
(5) Then, features of the head are obtained from the three-dimensional shape of the head and the mat position, and the features are used to estimate the head circumference using a machine learning algorithm.
(6) In developing and validating this system, we collected a total of 184 ideal images of newborns from the NICU, and those data were used to determine the system’s performance. This study introduces a non-contact method for measuring head circumference and demonstrates its potential to provide significant benefits for both the neonate and the healthcare professional.

## 2. Difficulties in Manual Head Circumference Measurement in Incubators

**Figure 1:**
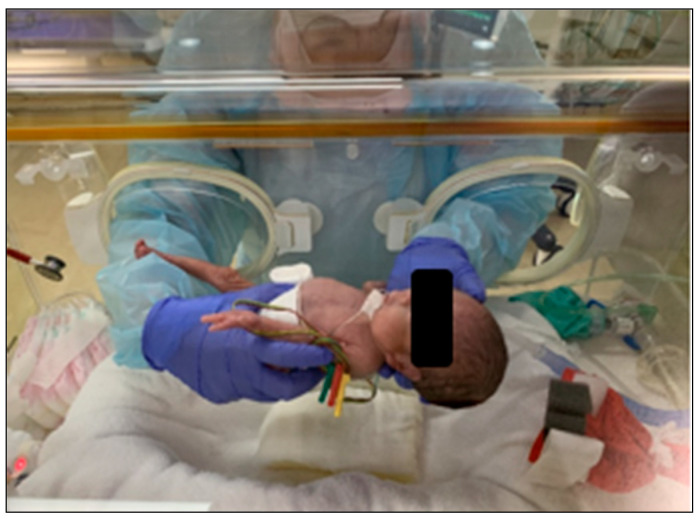
Newborn in NICU.

Generally, the standard birth weight for newborns is around 3000 g. However, some newborns are born with significantly lower weights, often below 1000 g, classifying them as low-birth-weight newborns. Due to their delicate nature, these newborns require specialized medical attention and are cared for in incubators until they are strong enough to thrive outside them. Incubators provide a controlled environment with stable temperature and humidity, which helps to regulate low-birth-weight newborns’ body temperatures, protect them from infections, and reduce stress on their developing systems. These controlled conditions are crucial for newborns who need specialized care and treatment. A low-birth-weight newborn in an incubator is shown in Figure 1.

However, these incubators make it difficult for healthcare providers to take care of newborns because they limit access to the newborns. Their enclosed design makes it difficult to measure growth. Additionally, healthcare providers need to carefully open, close, and manage their work in the incubators with minimal discomfort to the newborns. Therefore, extra precautions must be taken before, during, and after manual measurements, increasing the workload and measurement time. Although head circumference measurement is important for early healthcare assessment, traditional soft tape measurements are not ideal inside incubators, where the restricted space and limited accessibility make it difficult to obtain precise measurements.

## 3. Materials and Methods

### 3.1. Measurement System

**Figure 2:**
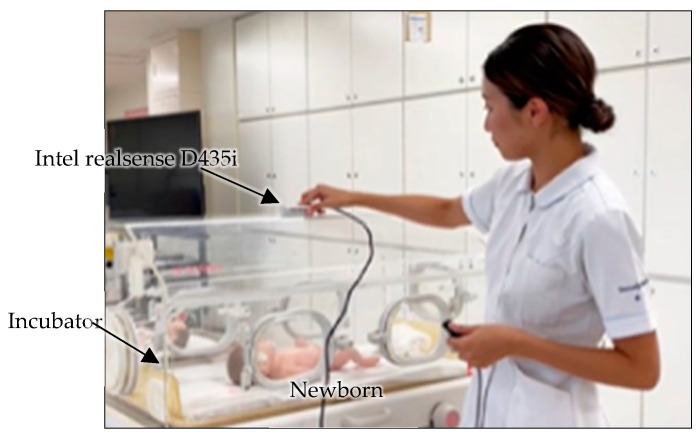
Measurement system.

The 3D measurement system used for head circumference assessment in the NICU is shown in Figure 2. The system consists of a 3D imaging sensor (Intel RealSense D435i, Intel Corporation, Santa Clara, CA, USA) and a laptop PC (Microsoft Surface Pro 9 with a 12th Gen Intel^®^ Core™ i7-1255U processor and 16 GB of LPDDR4x RAM, Microsoft Corporation, Washington, DC, USA) and allows head circumference measurement using non-contact one-shot images from outside the incubator. The Intel RealSense D435i is an RGB-D camera capable of capturing red, green, and blue (RGB) color data along with depth information simultaneously in a single frame. Because the depth information is integrated with the color stream, it enables the 3D reconstruction of the image scene for measuring newborns’ shapes. To increase measurement accuracy, the Intel RealSense D435i has already been calibrated for intrinsic and extrinsic parameters to reduce the distortions. Moreover, the RealSense D435i camera is resistant to outdoor lighting conditions, making it suitable for both indoor and outdoor use. This ensures that lighting variations, whether bright or dim, have no impact on its performance in different NICUs.

The one-shot image can be acquired in 1/30th of a second, eliminating the need to account for the newborn’s movements during the measurement process. The RGB-D sensor captures 16-bit depth images with a resolution of 640 × 480 pixels. These high-resolution depth data are sufficient for capturing the specific shape of the newborn.

In this setup, the incubators are constructed from transparent materials, which can potentially reflect light and interfere with the 3D imaging process. To solve this problem, the ability of the 3D imaging sensor to detect infrared light is utilized. A filter that absorbs visible light (CLAREX NIR-85N, Nitto Jushi Kogyou Co., Ltd.,Tokyo, Japan) is attached to the lens position of the infrared sensor. This approach helps to minimize reflections that could distort the data collected by the sensor, ensuring that the captured images provide accurate measurements of the newborn’s head circumference.

### 3.2. Coordinate Systems

**Figure 3:**
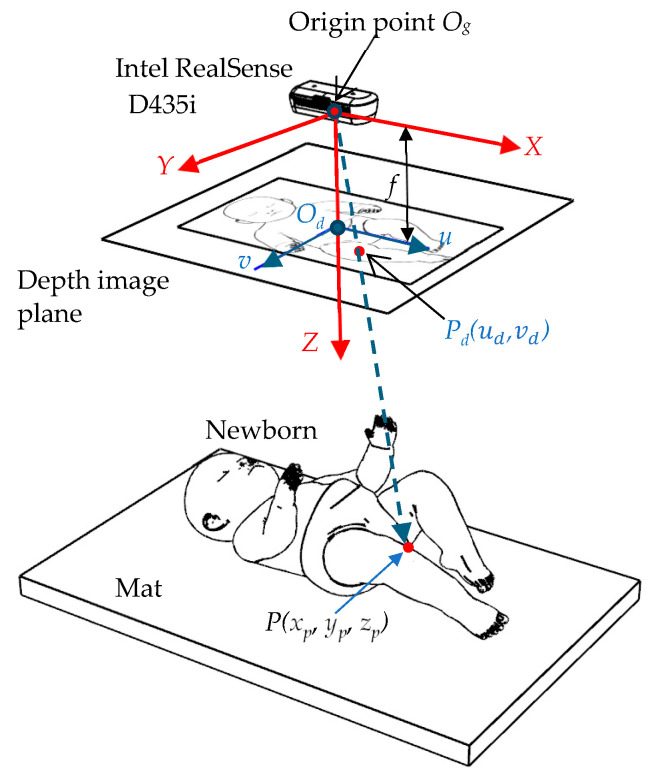
Coordinate systems of measurement setup.

The coordinate systems of the measurement setup are shown in Figure 3. The origin *O_g_* of the global coordinates (*x*, *y*, *z*) is set to the camera position. The RGB-D camera generates the depth image with the color image. The coordinate system of the depth image plane is (*u*, *v*) with the origin $O_{d}$ at the intersection with the global coordinate z-axis. The distance $z_{p}$ from the origin point *O_g_* is recorded at ($u_{d}$, $v_{d}$) in the depth image. The depth image plane can be assumed to be at a distance *f* from the origin point *O_g_*. A point ($x_{p}$, $y_{p}$, $z_{p}$) on the neonate surface is represented in these coordinate systems [28] as follows:

(1) $$x_{p}=-\frac{z_{p}}{f}\;u_{d}$$

(2) $$y_{p}=-\frac{z_{p}}{f}v_{d}$$

where $u_{d}$ and $v_{d}$ are points on the depth image, and $z_{p}$ can be obtained from the depth image.

**Figure 4:**
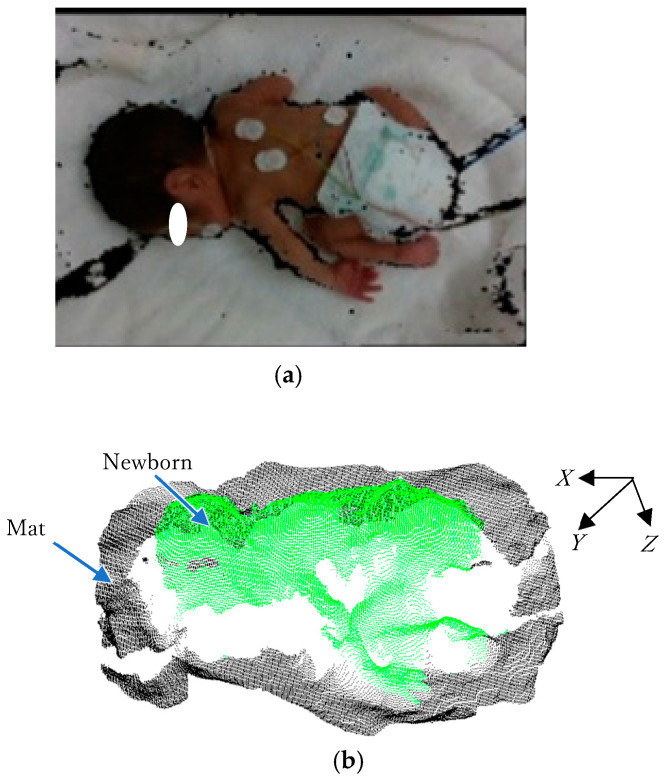
Conversion of 3D data. (**a**) Example of color image with depth image. (**b**) 3D transformation of depth image.

An example of a color image with a depth image captured by Intel RealSense D435i is shown in Figure 4a, and the converted 3D point cloud data from the depth image are shown in Figure 4b.

### 3.3. Flow of Head Circumference Estimation

**Figure 5:**
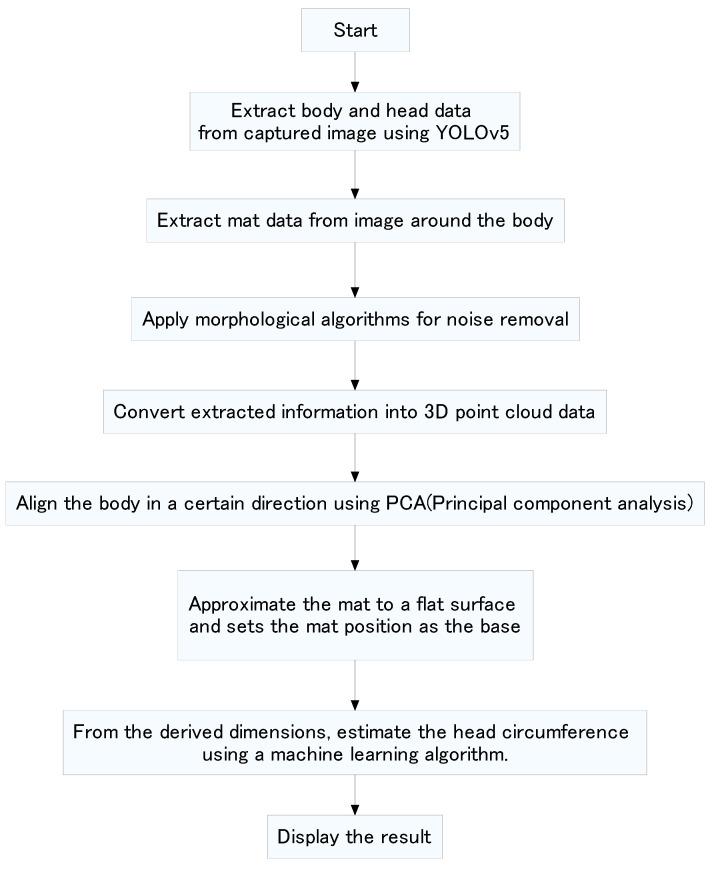
Processing flow.

Figure 5 shows a flow chart for head circumference estimation. The body and head are extracted from the captured image using YOLOv5 [29,30]. The data of the mat are also extracted from the extracted body perimeter. To reduce small noises in the 2D extracted image, a morphology algorithm is applied to remove isolated pixels and refine the object boundaries [31]. This algorithm efficiently minimizes the effects of unwanted artifacts that interfere with object segmentation. The morphology operation systematically reduces noise, ensures more accurate feature extraction, and ultimately improves the overall reliability of the measurement process. The extracted information is converted into three-dimensional point cloud data. Because the orientation of the camera and the posture of the newborn change slightly, PCA (principal component analysis) [32] is used to align the body orientation. Principal component analysis reveals the component with the greatest variation among the three-dimensional components of the point cloud data. The direction of the body with the largest variation is the orientation of the body, because the longitudinal direction of the body has the largest variation. In addition, the head positioning information is used to align the head to the left and the feet to the right. The mat surface is approximated as a plane, and this surface is used as the base. Considering the extracted head dimensions and the fact that the head is in contact with the mat, the head circumference is estimated using machine learning. The estimated results are displayed.

#### 3.3.1. Three-Dimensional Data of Head and Mat Extraction

**Figure 6:**
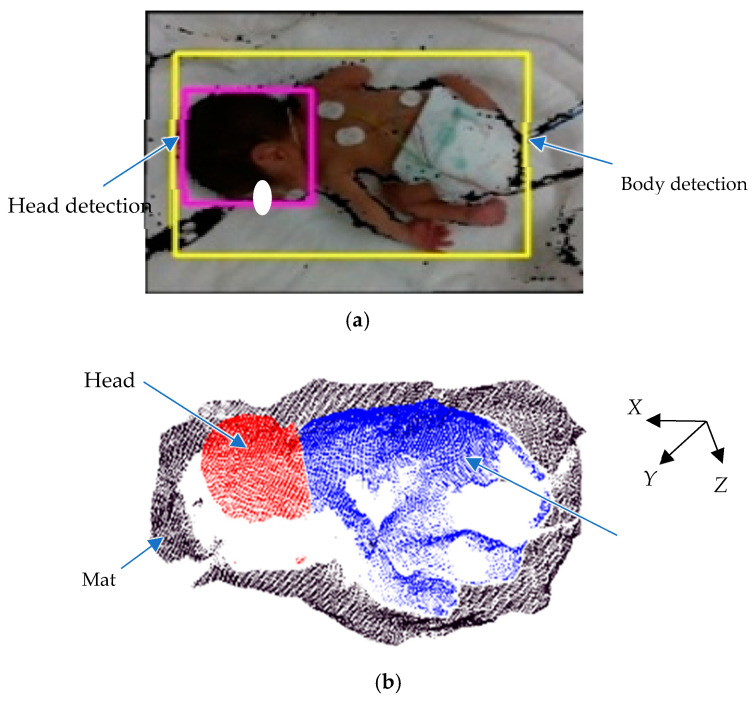
Head and mat extraction. (**a**) YOLOv5 extraction. (**b**) 3D data of extracted head and mat.

The first step in measurement is to extract the head and body in the captured image. In this system, YOLOv5 is employed to distinguish the head and body. Figure 6a illustrates the result of newborn detection using YOLOv5. The pink rectangle highlights the head detection results, while the yellow rectangle indicates body detection. Because YOLOv5 may include irrelevant parts of the newborn or background within the bounding boxes, the GrabCut algorithm [33] is employed to isolate the body and head from its background. The 3D output data of the GrabCut algorithm are shown in Figure 6b. The area around the newborn’s body is detected as a mat.

#### 3.3.2. Feature Extraction

Because the image is captured from above, only the upper surface of the head is visible, making it difficult to obtain the complete 3D structure of the head. However, acquiring head measurements from those invisible portions is also essential for accurate measurement. These missing areas are estimated using mat information. In most NICUs, towels are often used on the bed; variations in towel thickness, folding patterns, and placement complicate the process, introducing potential inaccuracies in accurately calculating the mat’s location relative to the camera. The mat on which a newborn infant lies, such as a blanket, is not a flat surface. Because the mat’s positional information is crucial for head circumference estimation, it is important to detect it reliably, even when the mat is not flat. To overcome these issues, a plane equation with the least square method is utilized, providing a more stable and consistent method for horizontal 3D mat detection. Equation (3) is used to generate the horizontal 3D mat location in the global coordinates. To achieve this, RANSAC (random sample consensus) is used, as it effectively identifies geometric shapes from noisy data [34]. RANSAC is an iterative method for estimating a mathematical model from a dataset containing noise. This approach significantly improves the precision of measurements, enabling the determination of the mat’s correct position relative to the camera.

**Figure 7:**
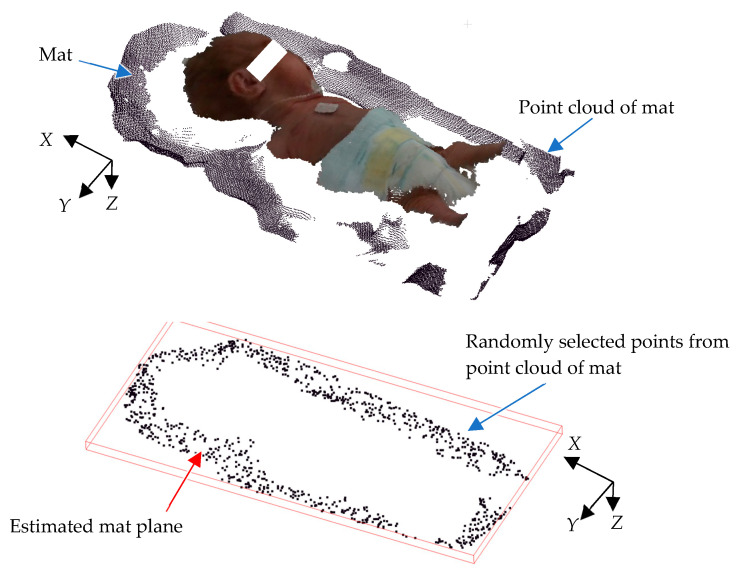
Mat plane estimation.

Figure 7 illustrates an example of 3D point cloud data for a mat. As shown, the data exhibit undulations due to the blanket’s shape. First, several data points are randomly sampled from the point cloud to derive a plane equation using Equation (3). The number of points near this plane is then counted to evaluate the plane equation’s consistency. A higher count indicates a better match between the plane and mat, as more point clouds align with the plane. Next, the plane equation is recalculated using a different randomly selected subset of mat data, and its consistency is re-evaluated using the same method. This process is repeated multiple times, and the plane with the highest evaluation value is determined to be the optimal representation of the mat’s surface.

*Ax* + *By* + *Cz* = 1 (3)

where $\left[\begin{array}{c} A\\ B\\ C \end{array}\right]={\left[\begin{array}{ccc} x_{1} & y_{1} & z_{1}\\ x_{2} & y_{2} & z_{2}\\ : & : & :\\ x_{n} & y_{n} & z_{n} \end{array}\right]}^{*}\left[\begin{array}{c} 1\\ 1\\ \begin{array}{c} :\\ 1 \end{array} \end{array}\right]$, * is a pseudomatrix, and (*x_n_*, *y_n_*, *z_n_*) are point cloud data of the mat.

In manual newborn head circumference measurement, the newborn is measured in the supine position. The head circumference is measured from the external occipital ridge through just above the right and left eyebrows. However, from outside the incubator, the baby’s position can vary from supine, face down, or sideways. To determine a stable measurement position, a spherical approximation using the least-squares method is used to determine the cross-sectional position from which the head circumference is estimated. Using the head data that can be obtained, the following equation in general form is used to determine the sphere approximation:

(4) $$({x-a)}^{2}+{\left(y-b\right)}^{2}+{\left(z-c\right)}^{2}=r^{2}$$

where center position of sphere is (*a*, *b*, *c*), and radius is *r*.

On the other hand, in standard form:

(5) $$x^{2}+y^{2}+z^{2}+kx+ly+mz+n=0\;\;\;\;\;$$

The center position (*a*, *b*, *c*) of sphere is

$$\left\{\begin{array}{c} a=-\frac{k}{2}\\ b=-\frac{l}{2}\\ c=-\frac{m}{2} \end{array}\right.$$

where

(6) $$\left[\begin{array}{c} k\\ l\\ \begin{array}{c} m\\ n \end{array} \end{array}\right]={\left[\begin{array}{ccc} x_{1} & y_{1} & z_{1}\\ x_{2} & y_{2} & z_{2}\\ : & : & :\\ x_{n} & y_{n} & z_{n} \end{array}\right]}^{*}\left[\begin{array}{c} -x_{1}^{2}-y_{1}^{2}-z_{1}^{2}\\ -x_{2}^{2}-y_{2}^{2}-z_{2}^{2}\\ \begin{array}{c} :\\ -x_{n}^{2}-y_{n}^{2}-z_{n}^{2}1 \end{array} \end{array}\right]$$

* is a pseudomatrix, and ($x_{n}$, $y_{n}$, $z_{n}$) are point cloud data for the head.

**Figure 8:**
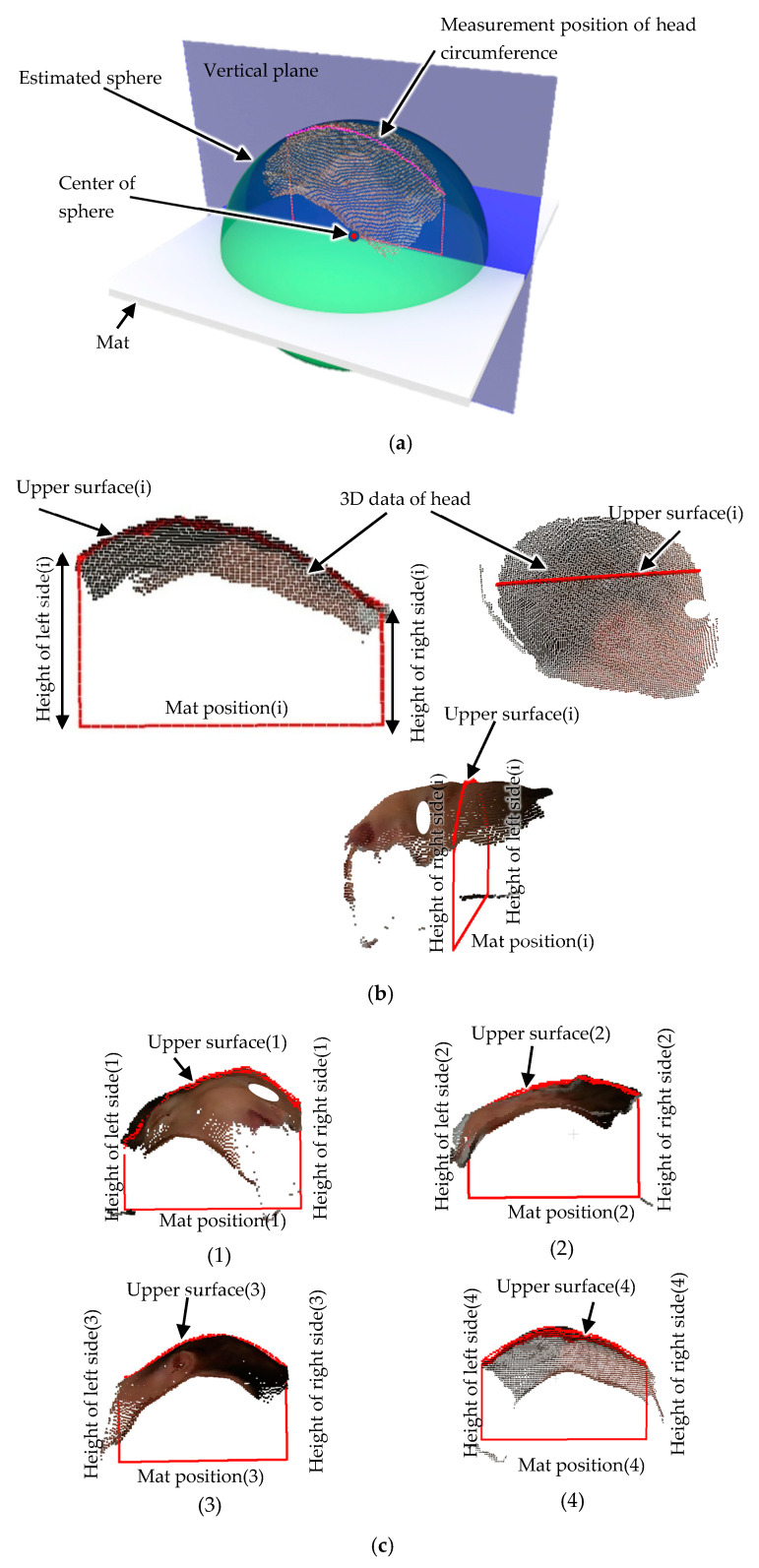
Feature extraction. (**a**) Head circumference location estimation using sphere. (**b**) A cross-section of the head at this vertical plane position. (**c**) Feature extraction using mat information.

The estimated sphere is superimposed on the point cloud data of the head in Figure 8a. The vertical plane passing through the center of the sphere was used as the measurement position for the head circumference to avoid it being affected by the orientation of the head. Figure 8b shows a cross-section of the head at this vertical plane position. In this figure, the bottom straight line indicates the mat position. Because the head is in contact with the mat, the head height can be calculated from the distance from the mat position. Because the images are taken from above the newborn, the shape of the head in contact with the mat and the left and right shapes of the head are not known. Therefore, head circumference estimation was performed using machine learning based on the height of the left and right sides of the head and the length of the upper surface of the head. These features are used in a random forest algorithm [35,36,37,38] to calculate the head circumference. Although there are parts of the head that cannot be seen because the images are taken from outside the incubator, if the head circumference can be estimated from the limited number of features that can be captured, the practicality of this method in the medical field can be demonstrated. In Figure 8c, feature extraction on different images is demonstrated.

## 4. Results and Discussion

### 4.1. Data Collection

The Perinatal Maternal and Child Health Center of University of Miyazaki Hospital is a comprehensive perinatal medical center with a 9-bed NICU for the management of newborns and a 12-bed Growing Care Unit. It also has two delivery rooms and three maternal–fetal intensive care units for the 24-h monitoring of high-risk pregnant women and fetuses. The center admits about 150 newborns each year, including about 30 very low-birth-weight babies weighing less than 1000 g. The center also provides risk management for morbid newborns, who may weigh less than 500 g. In this NICU, data on the physical measurements of newborns are collected. Because the center manages newborns from birth to discharge, there were no obstacles to collecting data on newborns’ physical measurements for experiments.

Ethical approval was obtained before data collection. The dataset consists exclusively of underweight newborns, and the collection period spanned from 20 September 2023 to 15 May 2024. Because NICU environments require strict safety measures and controlled access, direct and frequent physical entry is limited to authorized medical personnel. Therefore, to minimize unnecessary human presence and reduce the risk, the data collection process was conducted within the NICU, while subsequent data processing and analysis were carried out in a separate laboratory. When handling information related to the implementation of the research, the information was anonymized and managed with a research number that was unrelated to the personal information of the research subjects, so that it could not be immediately identified which research subject’s information was being handled. The correspondence list was stored on a computer disconnected from the network, and a password was set for the file, which was managed by the information manager.

This separation between data acquisition and processing necessitated the implementation of a remote-control system, which allowed efficient data transfer and communication between the NICU and laboratory. By leveraging remote connectivity, real-time data could be accessed, system performance could be monitored, and software configurations could be updated as needed without requiring direct physical access to the NICU. This approach not only ensured a seamless workflow but also contributed to maintaining a safe and sterile medical environment for the newborns. Additionally, the remote access system played a crucial role in enhancing the overall efficiency of the study by reducing potential disruptions in data collection while allowing us to continuously analyze the performance of the system.

### 4.2. Dataset Preparation for YOLOv5

**Figure 9:**
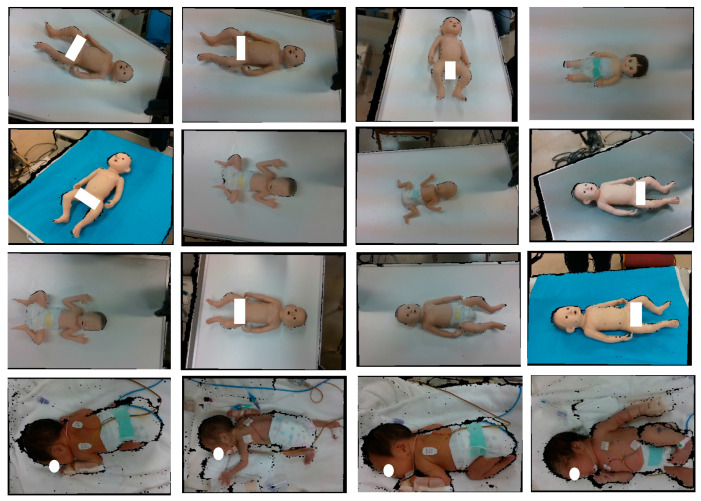
Dataset preparation.

The dataset used for YOLOv5 model development is shown in Figure 9. Although the bodies of newborns are usually in the same orientation in the incubator, doll models in various orientations were intentionally added to the training data to increase the robustness of recognition. For body detection, 50% of the data were obtained using model baby objects, while the remaining 50% were collected from the NICU; for head detection, 20% of the data were obtained using model baby objects and 80% of the data were taken from the real newborn dataset from the NICU. The use of model baby objects allowed for capturing a variety of lighting conditions, different backgrounds, and different angles to enhance the model’s usability in different real-world applications. Meanwhile, the real newborns’ data represent the original body color and natural body and head poses; this information is important for the actual newborns’ body and head detection. As access to the data from the NICU is limited, a combination of model baby objects and the real newborn dataset from the NICU data ensured a more robust and reliable dataset for developing the YOLOv5 model because YOLOv5 model development requires a large dataset for effective generalization; real newborns’ data alone are insufficient.

### 4.3. Training and Testing for YOLOv5 Process

For the body detection, a total of 2329 images with combination data were used, and these were divided into training data (2133 images) and testing data (196 images). For the head detection, a total of 1335 images with combination data were used for YOLOv5 model development; the training data were 1099 images, and the testing data were 236 images. The images were annotated using the MakeSense AI web application (December 2024). To minimize inconsistencies and errors in the ground truth labels, annotation was performed manually by labeling each body and head location in the images. This process ensured the precise detection of newborns, improving the accuracy and reliability of the YOLOv5 model.

The training was conducted on an NVIDIA RTX A5500 GPU (NVIDIA Corporation, California, USA) with a batch size of 16 over a total of 300 epochs. During training, the model was optimized using the optimizer with a learning rate scheduler to enhance convergence and prevent overfitting. For inferences of both models, the weights and biases were converted separately to ONNX format, and the exported ONNX model (YOLOv5) was utilized in C++ with OpenCV 4.5.4 for target body and head detection. The performance was evaluated using the mean average precision (mAP) metric; the mAP score for the testing data was 98% for body detection and 99% for head detection (Equation (7)). These results demonstrate the model’s ability to accurately detect newborns’ body and head positions, making it suitable for real-world applications in neonatal care.

(7) $$mean\;Average\;Precision\;=\;\frac{1}{n}\sum_{i=1}^{n}AP_{i}$$

where n is the total number of classes, and $AP_{i}$ is the average precision for each class. In this case, AP is computed from the area under the precision–recall (PR) curve. For detection, the intersection over union where the predicted bounding boxes overlap with the ground truth bounding boxes is used.

The intersection over union (IoU) finds major use in object detection; it measures how much a predicted bounding box coming from body and head detection overlaps with the ground truth bounding box annotated manually (Equation (8)). The IoU was particularly useful in this study because the precise localization of the newborn’s head is critical for accurate circumference measurement [39,40].

(8) $$IoU=\frac{Area\;of\;Overlap}{Area\;of\;Union}$$

where Area of Overlap represents the intersection between the predicted bounding boxes for body and head detection, while Area of Union represents the total area covered by all the bounding boxes detected using the developed body and head YOLOv5 models, minus the overlap area in the detection results.

### 4.4. Experimental Results

**Figure 10:**
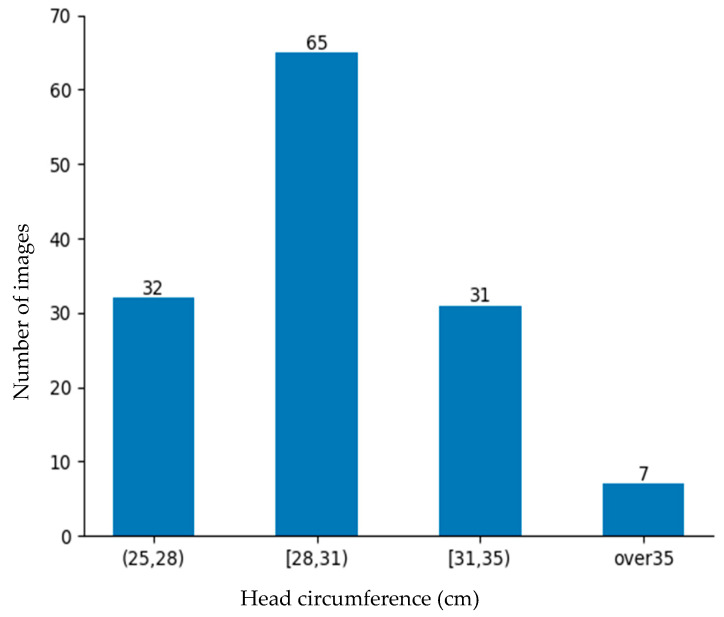
Training data.

**Figure 11:**
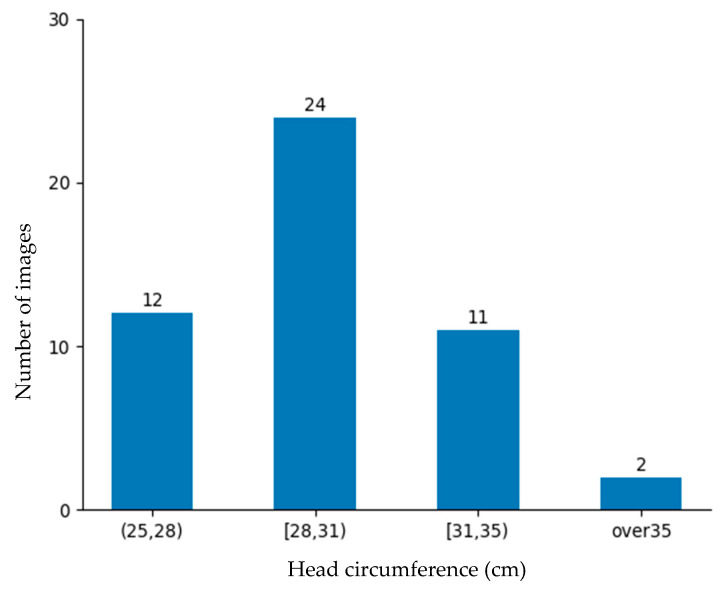
Testing data.

The rate of low-birth-weight infants is lower than that of normal-weight newborns. Obtaining data on low-birth-weight newborns is difficult due to their lower occurrence and the strict conditions required for data collection. Despite these barriers, 184 images were collected from the NICU. Although a large dataset is better, this dataset was deemed sufficiently representative for evaluating the proposed approach and demonstrating its practical feasibility in a rigorous neonatal healthcare environment. These data were divided into training data and testing data as shown in Figure 10 and Figure 11 for the machine learning-based head circumference estimation system.

In this system, manual measurement data obtained using a soft tape measure served as the ground truth data. The training data were used to train the model by making it learn features that correlated with the ground truth circumference. To evaluate the performance of the model and its generalization ability, the model was evaluated using test data prepared separately. Common regression metrics include the mean absolute error (MAE), mean squared error (MSE), and root mean squared error (RMSE). However, the MSE and RMSE are highly sensitive to outliers, which can significantly distort the evaluation results. In head circumference measurement, newborns’ head and body shapes vary due to natural movement, positioning, and anatomical differences. Consequently, relying solely on the MSE or RMSE may lead to misleading performance assessments. The MAE is preferred because it measures absolute differences without overemphasizing extreme values, providing a more stable and interpretable evaluation in centimeters. Therefore, the accuracy was defined using the mean absolute error (MAE) presented in Equation (9). The average MAE for the testing dataset (comprising 49 images) was calculated.

(9) $$Mean\;Absolute\;Error=\frac{1}{n}\sum_{i=1}^{n}\left|y_{i}-\hat{y_{i}}\right|$$

where *n* is the number of testing images, $y_{i}$ is the measurement results obtained using soft tape, and $\hat{y_{i}}$ is the estimation results obtained using the proposed system.

To assess the regression model’s feasibility, we implemented and evaluated tree-based algorithms, including decision tree, extra trees regressor, AdaBoost, and random forest regressor. Table 1 shows a comparison between the algorithms. Among these, random forest regressor achieved the lowest MAE (0.91 cm), demonstrating the most reliable performance. Therefore, it was selected for head circumference estimation in our system. This result demonstrates the model’s effectiveness in estimating head circumference with a relatively small error margin, highlighting its potential for practical applications.

**Figure 12:**
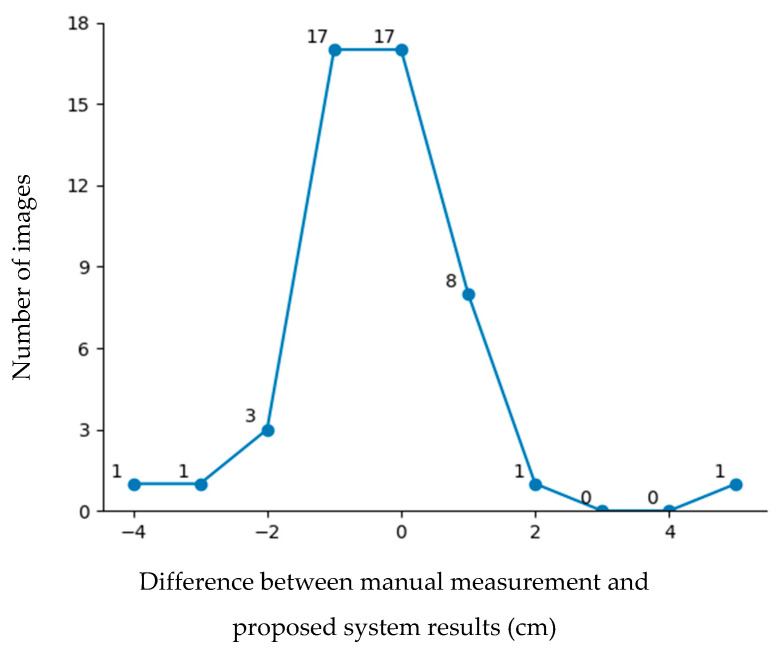
Error distribution.

Figure 12 shows the error distribution results for the testing data. According to the figure, nearly 86% of the images had an error of 1 cm or less. However, some images exhibited errors greater than 2 cm. Errors exceeding 2 cm were mainly due to two factors: data limitations and head orientation. The first issue stemmed from machine learning bias and an imbalanced dataset, particularly for head circumferences below 25 cm and above 35 cm, which were less represented in the training data because head circumferences below 25 cm and above 35 cm are rarely found in incubators. The second issue arose when the newborn’s head was significantly tilted or sloped, leading to measurement inaccuracies. To address these challenges, future work will focus on head direction adjustment to improve the measurement precision.

#### Comparison with Other Methods of Measuring Head Circumference

The comparison results for newborn head circumference measurements using our proposed system, a smartphone-based imaging system [16], and manual measurement are presented in Table 2. Our system utilizes a three-dimensional imaging system with an RGB-D camera for non-contact measurement. In contrast, manual measurement requires direct contact with the newborn, and the 2D smartphone imaging system, while reliable, requires calibration before measurement and may involve partial contact. As shown in Table 2, our proposed system offers the most reliable measurements without causing stress to either newborns or healthcare providers.

## 5. Conclusions

Although a quantitative evaluation is difficult, a questionnaire survey was conducted among the healthcare staff in the NICU of the University of Miyazaki, Japan, to gather the opinions of healthcare providers on the development of a non-contact measurement device using cameras. In this survey, 140 healthcare providers participated, and 99% of them agreed that it was necessary to develop a non-contact measurement device. Improvement of stress in nursing work will continue to be revealed in the survey.

Based on this survey, this paper introduces a method for measuring the head circumferences of newborns in the NICU using a 3D imaging sensor. To cope with occlusion and incomplete data from 3D image processing, this system uses mat information to estimate the invisible parts of the head and extract features. These features are then processed by a machine learning algorithm, enabling robust head circumference estimation. The results demonstrate reliable accuracy, with errors of approximately 1 cm or less in most typical cases, making the system suitable for practical use. However, some cases exhibit larger error margins. These errors are primarily attributed to the lack of sufficient data for very small newborns or those who do not need to be in incubators, which are rare in actual medical practice, and the head orientations of the newborns in the captured images. These issues with the data could affect the model’s performance and reliability. Despite these challenges, the system showcases its potential as a valuable tool for non-contact and efficient head circumference measurement, contributing to improved neonatal care and monitoring.

## 6. Future Work

In addition to head circumference, body length and weight are essential parameters for monitoring the growth and overall health of newborns. These measurements provide critical insights into neonatal development and help healthcare professionals to assess potential health risks. Therefore, this non-contact system using a 3D imaging camera for measuring body length and weight is intended to be used in future work for neonatal healthcare development. Expanding the system to include these additional measurements will enhance its clinical applicability, allowing for comprehensive growth monitoring without direct physical contact. This advancement aims to improve accuracy, reduce stress for both infants and medical staff, and contribute to the development of a fully automated neonatal assessment system in NICUs.

## Figures and Tables

**Table 1:** Performance comparison of regression models based on MAE.

Machine Learning Algorithm	MAE (cm)
Decision Tree	2.26
Extra Trees Regressor	1.15
AdaBoost	1.32
Random Forest Regressor	0.91

**Table 2:** Comparison between different methods of measuring head circumference.

	Manual Measurement	Our Proposed Method	Smartphone Imaging System
**Measurement method**	Traditional soft tape measurements.	Three-dimensional image processing using RGB-D camera.	Two-dimensional image processing using smartphone.
**Preprocessing and preparation**	Requires set up and preparation before measurement.	Immediate processing without additional steps required.	Some adjustments are necessary for calibration.
**Stress to healthcare provider and newborn**	Physical contact with the newborn is required.	No physical contact with the newborn and no management inside the incubator.	Some contact is required, though it is less stressful compared to the traditional method.
**Processing time**	It takes longer to perform and is dependent on the healthcare provider’s skill and experience.	Fast and efficient, allowing quick measurements with 2 s of processing for each image.	The processing time is not fully outlined, but it can take longer due to the additional time required for calibration.
**Cost**	Relatively low-cost equipment but may incur long-term costs due to errors and repeated measurements.	Initial investment is higher, but it offers long-term benefits.	Smartphones can be a cost-effective option.

## Data Availability

The datasets generated and analyzed during the current study are not publicly available due to neonate confidentiality and ethical restrictions. However, a portion of de-identified data may be available from the corresponding author upon a very strong reasonable request, subject to approval from the relevant ethics committee and compliance with institutional guidelines.
